# A Sri Lankan girl with a new genetic variant in the *PKLR* gene causing pyruvate kinase deficiency: a case report

**DOI:** 10.1186/s13256-021-02972-6

**Published:** 2021-07-27

**Authors:** Ahalyaa Sivashangar, Lallindra Gooneratne, Barnaby Clark, David Rees, Saroj Jayasinghe, Claire Laas

**Affiliations:** 1 Department of Pathology, Faculty of Medicine, University of Colombo, 25, Kynsey Road, Colombo 8, Sri Lanka; 2 Department of Molecular Pathology, Viapath at King’s College Hospital, King’s College Hospital, London, SE5 9RS UK; 3 Department of Paediatric Haematology, King’s College Hospital, London, SE5 9RS UK; 4 Department of Clinical Medicine, Faculty of Medicine, University of Colombo, 25, Kynsey Road, Colombo 8, Sri Lanka

**Keywords:** Sri Lankan, Genetic variant, *PKLR* gene, Pyruvate kinase deficiency, Case report

## Abstract

**Background:**

Erythrocyte pyruvate kinase is expressed under the control of the *PKLR* gene located on chromosome 1q21. Pyruvate kinase catalyzes the final steps of the glycolytic pathway and creates 50% of the red cell total adenosine triphosphate. Pyruvate kinase deficiency is the commonest glycolytic defect causing congenital non-spherocytic hemolytic anemia inherited in an autosomal recessive trait in which homozygotes and compound heterozygotes are common. Over 200 mutations have been described in patients with pyruvate kinase deficiency. This case report identifies a new pathogenic variant in *PKLR* gene detected in a patient with severe pyruvate kinase deficiency.

**Case presentation:**

A Sri Lankan Sinhalese girl who developed neonatal anemia and jaundice within 24 hours of birth with mild hepatomegaly. She was from a nonconsanguineous marriage and had two siblings who had no hematological disorders. She had repeated admissions due to similar illnesses and at the age of 8 years was found to have pyruvate kinase deficiency associated with a novel homozygous pathogenic variant c.507+1delG in the *PKLR* gene.

**Conclusions:**

A novel genetic variant in *PKLR* gene, consistent with pyruvate kinase deficiency, was detected in a Sri Lankan girl. This genetic variant may be specific to the Asian population and requires further studies.

## Background

Mature erythrocytes lack mitochondria and are therefore reliant on glycolysis for adenosine triphosphate (ATP) generation. Pyruvate kinase (PK) catalyzes the final steps of the glycolytic pathway to create 50% of total red cell ATP [1]. Human PK is regulated through expression of four enzymes. Erythrocyte PK is restricted to the R-type isoenzyme and is generated by expression of the *PKLR* gene (transcript NM_000298.5, 3053 bp), located on chromosome 1q21.

Pyruvate kinase deficiency (PKD) is the commonest glycolytic defect causing congenital non-spherocytic hemolytic anemia inherited in an autosomal recessive trait in which homozygotes and compound heterozygotes are common [1]. Estimated prevalence of the disease among Caucasians is 51 per million population [1]. There are no data available on prevalence of PKD in Sri Lanka. Over 200 mutations have been described in PKD [1]. Most common are missense mutations, including c.1529G>A, (p.Arg510Gln) in the USA and Northern and Central Europe; c.1456C>T, (p.Arg486Trp) in Southern Europe; and c.1468C>T, (p.Arg490Trp) in Asia [1, 2]. Small deletions, insertions, and frameshift mutations are rare, and a few large deletions have been reported [2]. This report highlights a new pathogenic variant in the *PKLR* gene, not described previously, in a patient with non-spherocytic hemolytic anemia. Interestingly the propositus is homozygous for this genetic variant, although she is the product of unrelated parents.

## Case presentation

An 8-year-old Sri Lankan, Sinhalese girl, the third child of nonconsanguineous parents, was delivered by elective cesarean section at 37 weeks of gestation with the Apgar 1^8^, 5^9^, and 10^9^. Her prenatal history was uneventful with a birth weight of 2.13 kg and head circumference of 35 cm. There was no family history of anemia or other hematological disorders. She was found to be pale and have yellow discoloration of skin and sclera 2 hours after birth (*T* = 0), which was highly suggestive of hemolysis. Her blood pressure was 80/55 mmHg, pulse rate was > 100 beats per minute, and she was well hydrated. She had no fever or hypothermia. She did not have any dysmorphism, lymphadenopathy, or splenomegaly. A nontender hepatomegaly was just palpable. Examination of systems was normal including the absence of focal neurological manifestations and limb weakness. At the initial presentation, her hemoglobin (Hb) was 55 g/L with normal white cells (total white cell count 8.8 × 10^9^/L, neutrophils 60%, nucleated red cells, and lymphocytes 35%) and platelets (430 × 10^9^/L). Blood film showed anisopoikilocytosis with marked polychromasia. Reticulocyte count was 9.6%. Total bilirubin was 15.6 mg/dL with an indirect fraction of 13 mg/dL. Rest of her liver function and renal function tests were within normal limits. There was no laboratory evidence of intravascular hemolysis. Urinalysis showed increased urobilinogen. Screen for bacterial and viral infections was negative. Both the mother and child were A Rh positive, and direct Coombs test in both was negative. In addition, the mother was negative for antibody screening. The baby was given double volume exchange transfusion and phototherapy on suspicion of a possible rare blood group incompatibility, although direct Coombs was negative.

A month later (*T* = 1), she presented with symptoms of anemia, and her Hb had fallen to 45 g/L. She was screened for G6PD deficiency and was found to have a normal G6PD enzyme level, 6.60 µg/dL (normal range 4.60–13.5 µg/dL) with a normal Brewer test. The osmotic fragility test was also negative. High-performance liquid chromatography (HPLC) of hemoglobin revealed no evidence of β-thalassemia or an Hb variant at 4 months of age. This was consistent with genetic analysis examining the five common β-globin gene mutations in Sri Lanka causing β-thalassemia (IVSI-1G>A, IVSI-5G>C, Cd8/9, Cd41/42) and HbE (*HBB*:c.79G>A, p.Glu27Lys), none of which was detected in her DNA sample. Bone marrow biopsy was compatible with hemolytic anemia with increased iron stores. Pyruvate kinase enzyme activity and 2,3-bisphosphoglycerate level were not assessed as these investigations are not performed in Sri Lanka. She had repeated admissions due to similar clinical features. At the age of 8 years, Sanger sequencing of the *PKLR* gene was performed on a blood sample provided to collaborators at King’s College Hospital, London, who had established a diagnostic gene sequencing assay of the pyruvate kinase gene (*PKLR*). The child’s genetic test was performed without any incurrence of costs to the patient as Sri Lanka is a lower-middle-income country and the test was not available in Sri Lanka. The gene sequencing identified her to be homozygous for the c.507+1delG pathogenic variant, which deletes the first base of the intron after exon 4. The last codon of exon 4 is GGG (glycine) and is followed by GT in the intron, and it is the G base in the intron that is deleted. This variant therefore affects the canonical 5ʹ donor splice site. As the proceeding exon bases are also GGG, the donor splice site is predicted to be one base into the exon, and the transcript will remove the last G base, causing a frameshift in the protein sequence, predicted to be p.(Gly170Valfs*9). These abnormal transcripts are usually removed by nonsense-mediated decay. As this individual is homozygous, she does not produce any pyruvate kinase enzyme, which is consistent with the diagnosis of pyruvate kinase deficiency.

She did not receive any specific interventions for her hemolysis. She continues to have low-grade hemolysis for which she is on regular blood transfusions at 1–2-monthly intervals since the diagnosis was made at the age of 8 years. This has resulted in iron overload for which she is on deferasirox. She has been followed up monthly at the hematology clinic. Her weight and height were within the reference range [median + 1 standard deviation (SD) in weight and height growth curves] for her age, with no organomegaly. Since she was on regular blood transfusions, her cognitive, motor, social–emotional, and neurophysiologic development was age appropriate. Her bone age was not assessed. Her quality of life was reasonably good. Her activities of daily living was not affected by the disease. Her parents are also coping with her condition pragmatically.

## Discussion and conclusion

**Fig. 1:**
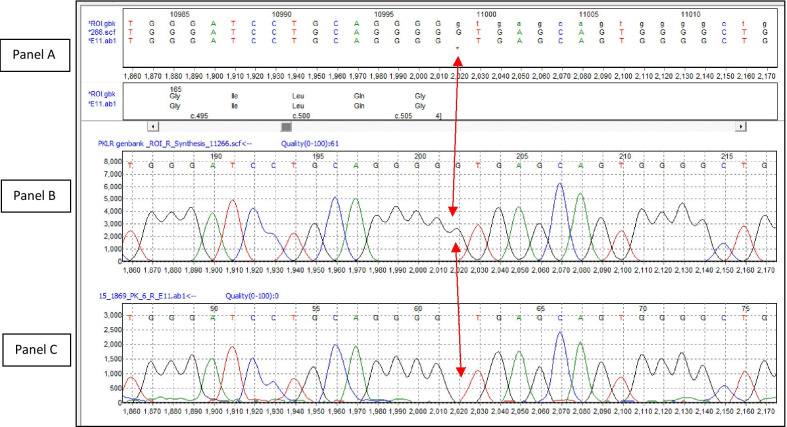
Sanger sequencing results from patient displayed in Mutation Surveyor software. **a** Genetic sequence where a run of five guanine (G) nucleotides in the reference sequence predicts a single G deletion represented by a black dot (highlighted by red arrow). **b** Predicted chromatogram that the software creates based on the reference sequence, showing a run of five G (black) peaks in the reference. **c** Chromatogram from the patient showing a homozygous deletion of one guanine (G) base, highlighted by the red arrow. The canonical splice site is represented by GT, which is at the end of the run of five guanine bases. The homozygous deletion of a G moves the splice site by one base, creating a frameshift in the amino acid coding sequence

This patient highlights a novel pathogenic variant (c.507+1delG) in the *PKLR* gene, which is consistent with non-spherocytic hemolytic anemia, most likely PKD. Interestingly, she was homozygous for the mutation (Fig. 1, gene sequencing) This is extremely rare since the mutation itself is the first such reported being absent from the Genome Aggregation Database (http://gnomad.broadinstitute.org/), which contains 125,748 exome sequences and 15,708 whole-genome sequences. Both parents were likely to be carriers; however, they could not be tested for the mutation, as it was not a test performed in Sri Lanka.

There are several reported cases of PKD with different pathogenic genetic variants in different populations. The first report was in 1961 in three patients with congenital non-spherocytic anemia [3]. A review in 2005 reports over 150 different mutations that have been identified [2]. Several genetic variants have been identified over the past 10 years, including a series of novel variants in Tunisians reported in 2017. To the best of out knowledge this is the first report of genetic variant specific to Asians. PKD is thought to protect against Malaria [4] similar to β-thalassemia and G6PD deficiency, and therefore, the existence of population-specific variants is likely.

This individual was homozygous for a novel pathogenic variant (c.507+1delG) in the *PKLR* gene, consistent with PKD. PK enzyme assay was not performed as it is not available in Sri Lanka, and therefore, PK deficiency could not be confirmed. However, her clinical presentation and blood film morphology were consistent with PKD. The exclusion of other etiologies for her clinical presentation further strengthens the diagnosis of PKD. Moreover, the genetic variant found in her *PKLR* gene is predicted to affect splicing of the mRNA transcript, and a pathogenic base substitution at the same locus has previously been reported as c.507+1G>A and abolishes the 5ʹ donor splice site [5] indicating that this locus is critical to normal *PKLR* gene expression. Historically, enzyme testing has been the gold standard for diagnosis, and *PKLR* gene testing has been performed mainly for confirmation or a research method. However, there are reports of falsely normal levels of PK enzyme in which genetic tests have been consistent with the clinical picture of PKD. In these cases, reticulocytes carrying other isoforms of pyruvate kinase give rise to increased levels of enzyme activity masking the effect of the genetic defect in the *PKLR* gene. Conversely, low PK enzyme activity has been identified in individuals who do not carry a *PKLR* gene defect. These individuals sometimes carry genetic variants in the *KLF1* gene, which encodes a transcription factor that drives expression of *PKLR* [6]. Therefore, genetic testing is essential for a definitive diagnosis and diagnostic in cases where enzyme assay results are equivocal [1].

In conclusion, this is a novel genetic variant in the *PKLR* gene, which is consistent with PKD, detected in a Sri Lankan girl, and has not been described previously. This variant may be specific to Asian populations and warrants further studies for confirmation.

## Learning points

- Searching for new genetic variants in the inherited disorders is important, as it will help to establish the diagnosis.
- Effective communication with the genetic laboratories is essential to establish the correct diagnosis, which helps to provide the best management for the patient.
- Reporting new genetic variants in international journals will help in the future diagnosis of the disease.

## Data Availability

Not available.

## Abbreviations

DNA: Deoxyribonucleic acid
G6PD: Glucose-6-phosphate dehydrogenase
PCR: Polymerase chain reaction

## References

[1] Grace RF, Zanella A, Neufeld EJ, *et al*. Erythrocyte pyruvate kinase deficiency: 2015 status report. Am J Hematol. 2015;90:825–30. 10.1002/ajh.24088 PMC5053227 26087744

[2] Zanella A, Fermo E, Bianchi P, *et al*. Red cell pyruvate kinase deficiency: molecular and clinical aspects. Br J Haematol. 2005;130:11–25. 10.1111/j.1365-2141.2005.05527.x 15982340

[3] Valentine WN, Tanaka KR, Miwa SA. Specific erythrocyte glycolytic enzyme defect (pyruvate kinase) in three subjects with congenital non spherocytic haemolytic anaemia. Trans Asssoc Am Physicians. 1961;74:100–10. 13924348

[4] Min-Oo G, Fortin A, Tam MF, *et al*. Phenotypic expression of pyruvate kinase deficiency and protection against malaria in a mouse model. Genes Immun. 2004;5:168–75. 10.1038/sj.gene.6364069. 15029238

[5] Wijk RI, Van Wesel AC, Thomas AA, *et al*. Ex vivo analysis of aberrant splicing induced by two donor site mutations in PKLR of a patient with severe pyruvate kinase deficiency. Br J Haematol. 2004;125:253–63. 10.1111/j.1365-2141.2004.04895.x. 15059150

[6] Viprakasit V, Ekwattanakit S, Riolueang S, *et al*. Mutations in Krüppel-like factor 1 cause transfusion-dependent hemolytic anemia and persistence of embryonic globin gene expression. Blood. 2014;123:1586–95. 10.1182/blood-2013-09-526087. 24443441

